# Analysis of Paint Properties According to Expandable Graphite and Fire Simulation Research on Firewall Penetration Part

**DOI:** 10.3390/polym16010098

**Published:** 2023-12-28

**Authors:** Seonghun Yu, Jonghyuk Lee, Donghyun Yeo, Junhee Lee, Jinseok Bae, Jeehyun Sim

**Affiliations:** 1 DYETEC (Dyeing & Finishing Technology Institute), Computer Aided Engineering (CAE) Center, Daegu 41706, Republic of Korea; enviro1234@dyetec.or.kr (S.Y.); trueleejh@dyetec.or.kr (J.L.); yd@dyetec.or.kr (D.Y.); junhee@dyetec.or.kr (J.L.); 2 Department of Textile System Engineering, Kyungpook National University, Daegu 41566, Republic of Korea

**Keywords:** fire simulation, virtual engineering, expandable graphite, mixed paint, fire wall penetration part

## Abstract

In this research, we attempted to develop paints that can be applied to various fields such as high-rise building structures and electric vehicle batteries. To minimize damage to life and property in the event of a fire, we attempted to manufacture a highly elastic paint material that can block flames and control smoke spread, and that has additional sound insulation and waterproofing functions. A high-elasticity paint was manufactured by mixing a flame-retardant polyurethane dispersion (PUD) with an acrylic emulsion binder and adding different mass fractions of expandable graphite (EG). The thermal, physical, and morphological properties of the prepared mixed paint were analyzed. The thermal properties of the mixed paint were analyzed and intended to be used as input data (heat transfer coefficient, specific heat capacity) for fire simulation. Output data were used to predict how much the temperature would change depending on the time of fire occurrence. The reason for conducting simulations on the fire stability of paint materials is that the fire stability of paints can be predicted without conducting fire tests. Two hours after the fire broke out, the thermal temperature distribution was analyzed. The temperature distribution was compared with and without mixed paint. Two hours after a fire broke out in a virtual space, it was found that when the mixed paint was applied, the surrounding temperature of the penetration area was lower than when the mixed paint was not applied. Development costs for developing excellent paints can be reduced. Since fire safety can be predicted without actually conducting tests, the time required for product development can be reduced. We are confident that this is a very groundbreaking technology because it allows fire safety simulations for developed products to be conducted in a virtual space by creating an environment similar to actual fire test standards.

## 1. Introduction

With the development of architectural design technology, the size of buildings has become larger. As the structure of buildings has become more complex, the number of people inside the buildings has increased. As the scale of electrical and electronic facilities in buildings has also become larger, the likelihood of death and property damage in the event of a fire has increased. Paint manufacturing technology to prevent fires needs to evolve in line with the development of architectural design technology.

Most paints used to improve flame retardancy and fire safety are halogen-based (Br, Cl, etc.) flame retardants. When paint is burned, it is subject to regulation in developed countries such as the United States, Germany, and France due to the generation of toxic gas (HBr) or carcinogens (dioxine). To replace existing paints, non-halogen paints such as inorganic, phosphorus, and melamine paints are used. However, they are mostly being used as an additive to combustible substances [1,2,3,4,5]. In accordance with global eco-friendly trends and regulations on flame retardant performance, research and development on paint materials is being conducted. In particular, more emphasis is placed on the development of paints with lower heat transfer coefficients than existing products [6,7,8,9].

Zhu [10] and others conducted research to improve the total heat release and tensile strength of non-halogen paints. Zhu and others conducted a study on adding 0.2 to 0.5% of expandable graphite to an acrylic copolymer filled with an alkaline compound, amorphous silica, or inert gas. The results showed that when the mass fraction of expandable graphite was 0.3 wt.%, the total amount of heat release was reduced the most. Kim [11,12,13] et al. conducted research on a paint composition with excellent waterproofing and insulation performance by adding 0.5 to 1 wt.% of expandable graphite to a mixture of polyvinyl alcohol-based compounds, acrylic monomers, and water-soluble polyurethane. When the mass fraction of expandable graphite was 0.5 wt.%, the insulation performance showed an improvement of more than 12.5% compared to when expandable graphite was not added.

In order to manufacture a paint material with excellent flame retardancy and fire stability using non-halogen-based materials, the addition of expanded graphite is essential. Since the physical properties and thermal stability of the paint material change greatly depending on the mass fraction of expanded graphite, the mass fraction of expanded graphite is a very important factor in manufactured non-halogen-based paints [14,15,16,17]. Acrylic resin has excellent heat resistance and excellent wear resistance. Additionally, because the drying time is short, the work can be completed quickly. When mixing expanded graphite with acrylic resin, performance such as fire prevention performance, wear resistance, and heat resistance can be secured.

In this study, we attempted to develop paints that can be applied to various fields such as high-rise building structures and electric vehicle batteries. To minimize damage to life and property in the event of a fire, we attempted to manufacture a highly elastic paint material that can block flames and control smoke spread, and that has additional sound insulation and waterproofing functions. A highly elastic paint material was manufactured by mixing an acrylic emulsion binder with a flame-retardant polyurethane dispersion (PUD) and adding different mass fractions of expandable graphite (EG). The thermal, physical, and morphological properties of the mixed paint were analyzed. After analyzing thermal, physical, and morphological properties, the best paint was selected. The fire stability of the paint material was predicted by conducting a simulation with and without applying the paint material to the area called the “firewall penetration part” of the building’s internal structure. At this time, a 3D model was created using the thermal property database of the paint as input data. Output data were used to predict how much the temperature would change depending on the time of fire occurrence. The reason for conducting simulations on the fire stability of paint materials is that the fire stability of paints can be predicted without having to manufacture actual building samples and conduct fire tests, which can reduce the development costs for developing excellent paints. Since fire safety can be predicted without actually conducting tests, the time required for product development can be reduced. We are confident that this is a very groundbreaking technology because it allows fire safety simulations for developed products to be conducted in a virtual space by creating an environment similar to actual fire test standards (ASTM E1966 [18], ASTM E814 [19]) in a virtual space.

## 2. Materials and Methods

### 2.1. Manufacture of Materials

**Figure 1:**
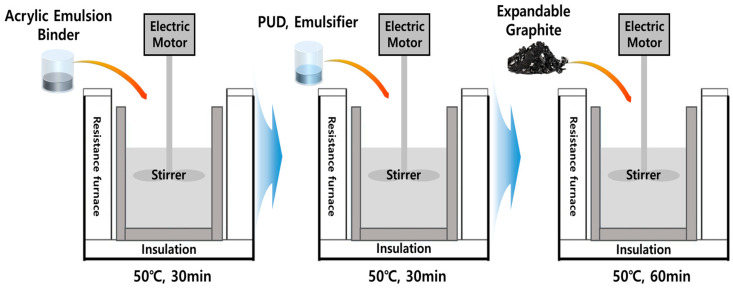
Schematic diagram of mixing process of high-elasticity paint material and thermally expansible graphite.

In this research, acrylic emulsion binder (SB-300, Kangnam jevisco Co., Ltd., Busan, Republic of Korea), polyurethane dispersion (PUD, GRP-064, T&L Co., Ltd., Anseong, Republic of Korea), Emulsifier (B25, Simgma-Aldrich, St. Louis, MO, USA), and expandable graphite (NA 23, Simgma-Aldrich, St. Louis, MO, USA) were used to prepare a mixed paint. The thermal properties of the manufactured mixed paint were analyzed and intended to be used as input data for fire simulation. Conditions for manufacturing mixed paints are shown in Table 1. The mass fraction of expandable graphite was set at 0.1, 0.3, 0.5, and 0.7 wt.%. The mass fraction of the acrylic emulsion binder was adjusted according to the mass fraction of expandable graphite. A schematic diagram of mixing an acrylic emulsion binder, polyurethane dispersion, emulsifier, and expandable graphite is shown in Figure 1. The basic properties of expandable graphite are shown in Table 2. The mixed paint material was cured for 24 h at a temperature of 23 ± 1 °C and humidity of 50 ± 5%, and specimens necessary for thermal stability and physical property analysis were produced.

### 2.2. Thermal Characterization

To analyze the thermal properties of the prepared mixed paint, the thermal conductivity coefficient and specific heat measurements were performed. The thermal conductivity coefficient test of the prepared mixed paint was conducted using a heat flux measurement (HFM, NETZSCH, Bobingen, Germany). The thermal conductivity coefficient was measured according to ASTM C117 [20] and calculated according to Equation (1).

(1) $$\lambda =QA(T_{H}-T_{C})/L$$

in which

λ = thermal conductivity coefficient, W/m·K;

Q = heat flux, W;

A = heat transfer area, m^2^;

T*_H_* = high temperature, K;

T*_C_* = low temperature, K;

L = thickness, m.

The specific heat of the paint mixed with expandable graphite was measured using a differential scanning calorimeter (DSC, Q500, TA Instrument, New Castle, DE, USA). Specific heat is an indicator of the amount of heat needed to raise the temperature of 1 g of a substance by 1 °C. Specific heat data are necessary for creating 3D models and simulating thermal properties of mixed paints. In order to measure specific heat with DSC, the enthalpy difference between the standard sample and the mixed paint sample must be obtained. The specific heat can be obtained by comparing the DSC curves of the standard sample and the mixed paint sample. The specific heat according to the mass fraction of expandable graphite was calculated according to Equation (2), shown in KS M ISO 11357-4 [21].

(2) $$C_{P}=\frac{h}{H}\times \frac{m^{\prime }}{m}\times C_{p}^{\prime }$$

in which

C_p_ = specific heat capacity of the test specimen, J/kg·K;

m = mass of test specimen, kg;

m′ = mass of standard material, kg;

C′_p_ = specific heat capacity of standard materials, J/kg·K;

h = the difference in the vertical axis direction of the DSC curve between an empty container and a container containing a test specimen, mW;

H = the difference in the vertical axis direction of the DSC curve between an empty container and a container containing standard substance, mW.

The flammability test of paint mixed with expandable graphite was conducted using a cone calorimeter (CC-1-X, Govmark, New York, NY, USA). The flammability test was conducted to measure the actual fire resistance of the mixed paint. The flammability test was conducted three times for each sample. The total heat release rate (THR), ignition, and mass reduction rate of mixed paint can be measured using cone calorimeter equipment. A cone-shaped heater was used. The test specimen was heated at a heat flux of 50 kW/m^2^. Flammability tests were conducted using an electric ignition source.

### 2.3. Mechanical Characterization

In order to analyze the physical properties of the prepared mixed paint material according to the expandable graphite mass fraction, tensile strength and elongation were analyzed according to ASTM D412 [22]. To manufacture a specimen according to ASTM D412, the mixed paint material was cured into a square shape of 200 × 200 mm and 3 mm thick. After cutting the cured specimen to size, a total of 5 specimens were manufactured. Tensile strength tests were conducted on the five manufactured specimens.

### 2.4. Morphological Characteristics Analysis

A field emission scanning electron microscope (FE-SEM, merlin compact, resolution 0.8 nm, Carl Zeiss, Oberkochen, Germany) was used to analyze the morphological characteristics of the mixed paint material according to the mass fraction of expandable graphite. The specifications of the equipment used to analyze the morphological characteristics according to the mass fraction of expandable graphite are as follows. The probe current is from 5 pA to 100 nA, and the acceleration voltage is from 0.02 V to 30 kV. The magnification is 12~200,000×. According to research results by Lee [23,24,25] and others, when adding inorganic materials to a polymer solution, thermal stability improves as the amount of inorganic materials added increases, but surface roughness improves due to agglomeration between inorganic materials. Accordingly, we attempted to analyze the correlation between the degree of particle aggregation and thermal properties by observing the surface of mixed paint materials according to the mass fraction of expandable graphite.

### 2.5. 3D Modeling and Boundary/Load Condition

**Figure 2:**
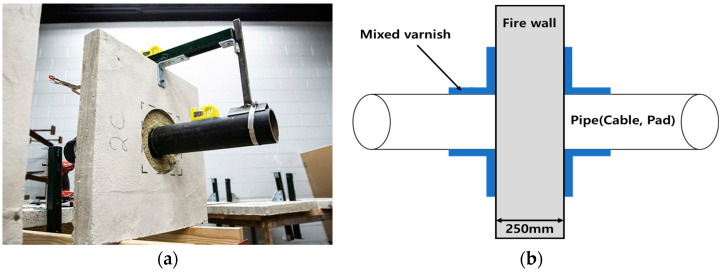
Actual view and simple model of the penetration part of the firewall. (**a**) Actual view of fire wall. (**b**) Simple model for simulation.

Firewalls, which are commonly found in large-scale buildings, must be constructed to be fireproof. In this study, we attempted to simulate fire stability in the “firewall penetration part” with and without mixed paint materials. A 3D model was created by applying data from the mixed paint material with the best thermal properties. The actual appearance of the penetration part of the firewall and a simple model for fire simulation are shown in Figure 2.

**Figure 3:**
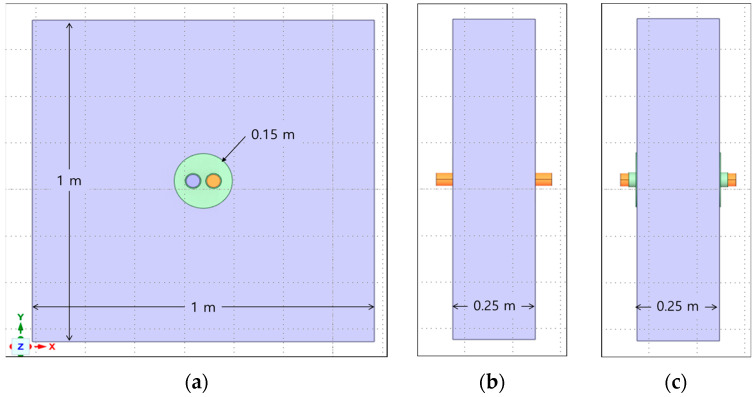
Fire model of the penetration part. (**a**) Fire model (front). (**b**) Fire model (side, without mixed paint). (**c**) Fire model (side, with mixed paint).

First, we attempted to implement the penetration part of the firewall as a 3D model in a virtual space. For 3D modeling, ANSYS (ANSYS 2022, Seattle, WA, USA), a commercial finite element analysis program, was used. A penetration part with a diameter of 150 mm was implemented in a firewall measuring 1 m × 1 m × 0.25 m. Inside the penetrating part are copper cables and PVC cables with a diameter of 40 mm, and the remaining space is occupied by urethane pads. The fire model for the firewall penetration area is shown in Figure 3. The penetration part of the firewall may have foreign substances or other dust on the surface. Because fire simulation depending on the presence or absence of mixed paint is important, it was assumed that other foreign substances did not exist in the penetration part of the firewall.

**Figure 4:**
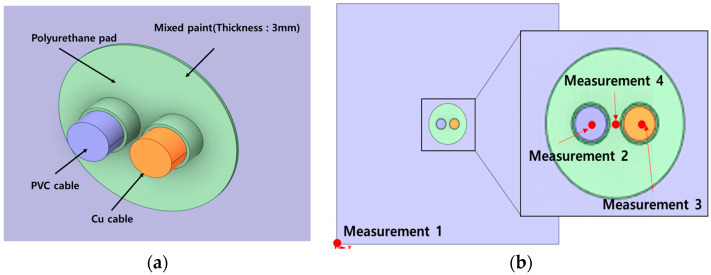
Detailed view of the penetration part. (**a**) Detailed view with mixed paint. (**b**) Measurement point (1~4).

The mixed paint material was applied to the front and back of the penetration area at a thickness of 3 mm. The fire stability was compared when the mixed paint was applied to the penetration area and when it was not applied to the penetration. To predict the fire safety of the penetration area, a simulation was conducted by setting the measurement points at the firewall wall (point 1), copper cable (point 2), PVC cable (point 3), and between the two cables (point 4). Figure 4 shows the mixed paint applied to a thickness of 3 mm and the fire stability measurements at four locations.

**Figure 5:**
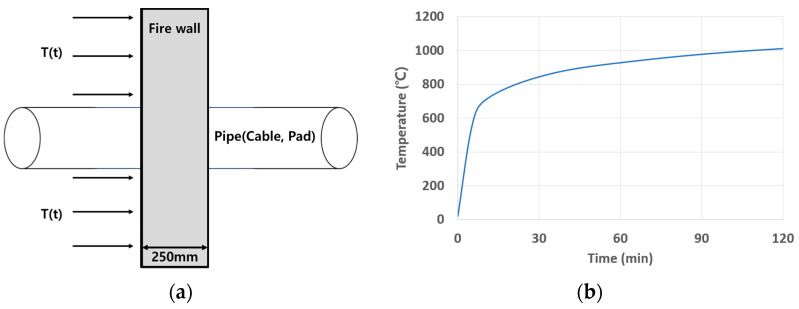
Boundary conditions and thermal load conditions for fire simulation. (**a**) Boundary condition. (**b**) ASTM E119 standard time–temperature curve.

The fire simulation conditions were set as follows. When a fire occurs in the penetration part of the firewall, the total time for which the fire occurs and the temperature according to the time the fire lasts are set as shown in Figure 5, in accordance with ASTM E119 [26].

## 3. Results and Discussion

### 3.1. Thermal Characterization Results

**Figure 6:**
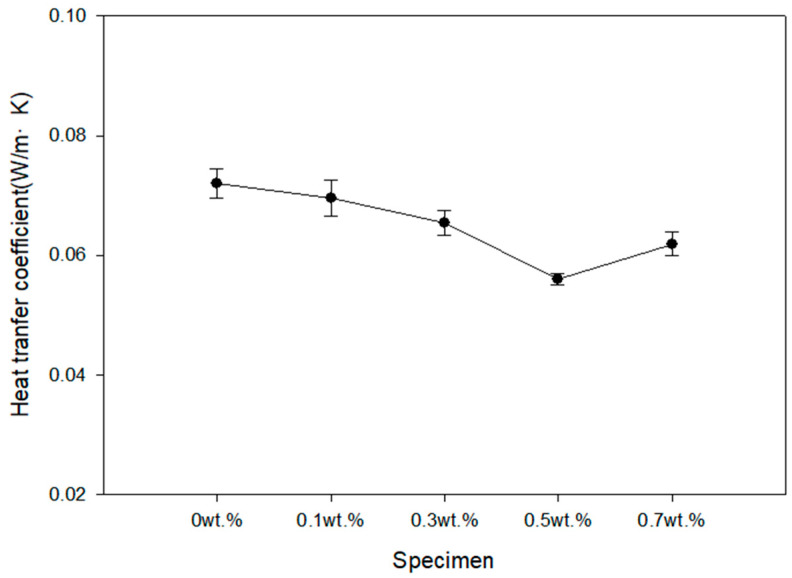
The thermal conductivity coefficient results according to the mass fraction of the expandable graphite.

The heat transfer coefficient results according to the mass fraction of expandable graphite are shown in Figure 6. When the mass fraction of expandable graphite was 0.5 wt.%, the relatively lowest heat transfer coefficient was shown. When the mass fraction of expandable graphite was 0.7 wt.%, the heat transfer coefficient increased by more than 10.5% compared to when the expandable graphite was 0.5 wt.%. When the mass fraction of expandable graphite becomes excessively high, agglomeration occurs between inorganic particles due to the van der Waals force, preventing the particles from being evenly distributed. When inorganic particles aggregate excessively, empty space increases, so it is believed that the thermal conductivity coefficient has increased sharply. The sample to which expandable graphite was not added showed an increase of 28.7% compared to the case where the mass fraction of expandable graphite was 0.5 wt.%.

**Figure 7:**
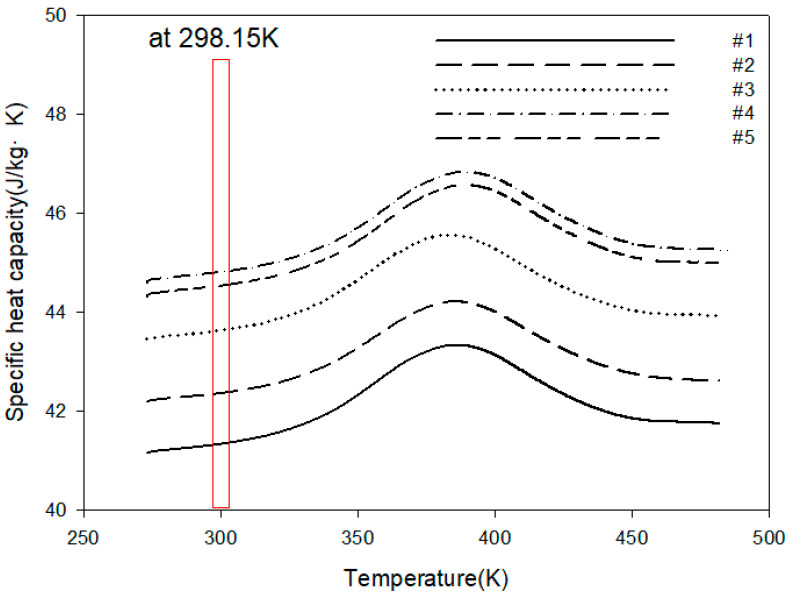
Specific heat capacity results according to the mass fraction of expandable graphite.

The specific heat results according to the mass fraction of expandable graphite are shown in Figure 7. Similar to the heat conduction coefficient results, the specific heat value was highest when the mass fraction of expandable graphite was 0.5 wt.%. However, when comparing the specific heat value when the mass fraction of expandable graphite was 0.7 wt.% and the specific heat value when the mass fraction was 0.5 wt.%, the approximate value was within ±5%. In the case of specific heat, it is judged not to be greatly affected by the agglomeration phenomenon between inorganic particles. However, if the expandable graphite is added excessively, it may cause adverse effects.

Table 3 shows the results of the analysis of total heat release rate (THR), ignition, and mass reduction rate of the mixed paint according to the mass fraction of expandable graphite. It was confirmed that no ignition occurred in all specimens. When measuring THR (10 min) of the mixed paint, the specimen with a mass fraction of expandable graphite of 0.5 wt.% showed the lowest value at 1.82 MJ/m^2^. The specimen with a mass fraction of expandable graphite of 0.7 wt.% showed a value of 1.87 MJ/m^2^. This is an approximate value within ±5%. When measuring the mass reduction rate of the mixed paint, the highest value was shown in the specimen without expandable graphite mixed. It was confirmed that the fire resistance of the paint was improved when expandable graphite was mixed. Through the flammability test of the mixed paint, it was confirmed that the fire resistance of the specimen with a mass fraction of expandable graphite of 0.5 wt.% was the best.

After building a cone calorimeter experimental environment in a virtual space, being able to simulate flammability tests on virtual samples can provide very interesting information to other researchers. We will continue to pursue this research. By using simulation technology, the cost and time required for actual fire resistance measurement tests can be reduced. In the event of an actual fire, paint with high fire resistance can reduce casualties and property damage. We also plan to conduct experiments on the fire resistance of paint in a large-scale fire environment.

### 3.2. Mechanical Characterization Result

**Figure 8:**
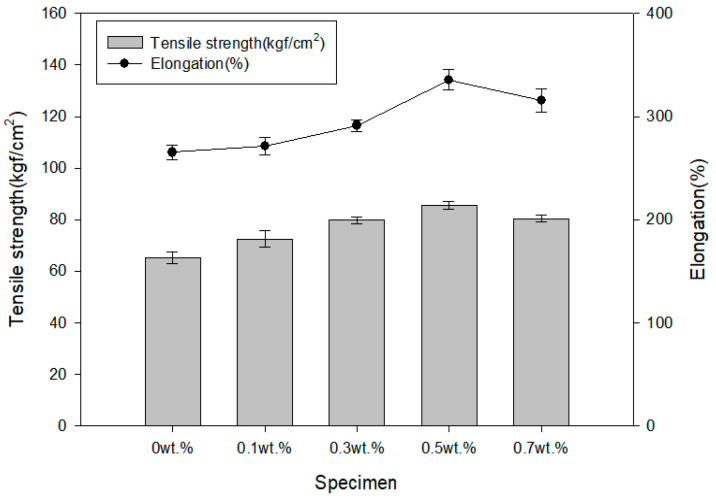
Results of physical property analysis according to mass fraction of expandable graphite.

The results of the analysis of tensile strength and elongation of the mixed paint according to the mass fraction of expandable graphite are shown in Figure 8. According to existing research results, when carbon nanotubes or graphite, which are inorganic materials, are added to polymer solutions or high-viscosity materials, physical properties are slightly improved [27,28,29].

When the mass fraction of expandable graphite was 0.5 wt.% or less, tensile strength and elongation tended to increase as expandable graphite was added. When the mass fraction of expandable graphite was 0.7 wt.%, the value decreased by 15.6% compared to when the mass fraction of expandable graphite was 0.5 wt.%. Similar to the results of the thermal property analysis, it is believed that agglomeration occurred between the expandable graphite particles, thereby reducing the physical properties.

### 3.3. Morphological Characteristics Results

**Figure 9:**
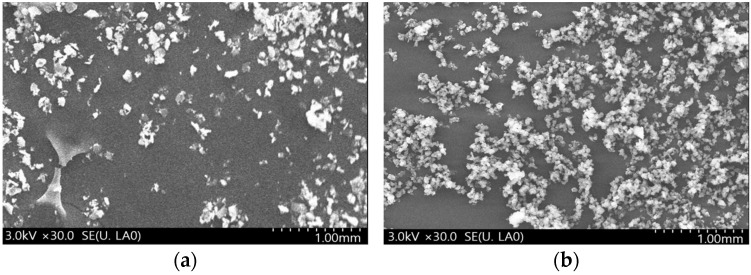

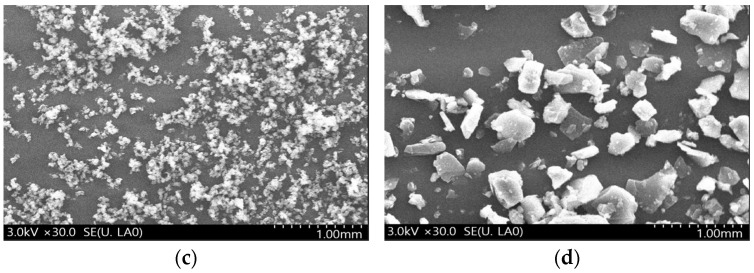
FE-SEM results according to EG mass fraction. (**a**) 0.1 wt.% (EG). (**b**) 0.3 wt.% (EG). (**c**) 0.5 wt.% (EG). (**d**) 0.7 wt.% (EG).

The results of the analysis of morphological characteristics of the mixed paint material according to the mass fraction of expandable graphite are shown in Figure 9. As a result of morphological characteristics analysis, the number of inorganic particles observed per unit area tended to increase as the mass fraction of expandable graphite increased. When the mass fraction of expandable graphite was 0.7 wt.%, the particles agglomerated due to agglomeration between particles. It was not evenly distributed and showed agglomeration. In the case of mixed paint samples where agglomeration occurred, thermal stability and physical properties were judged to be reduced. If the particles of expandable graphite can be distributed evenly, the thermal stability and physical properties of the mixed paint will be improved.

### 3.4. Fire Simulation Result

When the mass fraction of expandable graphite was 0.5 wt.%, the thermal and physical properties were the best, and the heat transfer coefficient and specific heat results were used as input data for analysis. The material properties used in the fire model are shown in Table 4.

**Figure 10:**
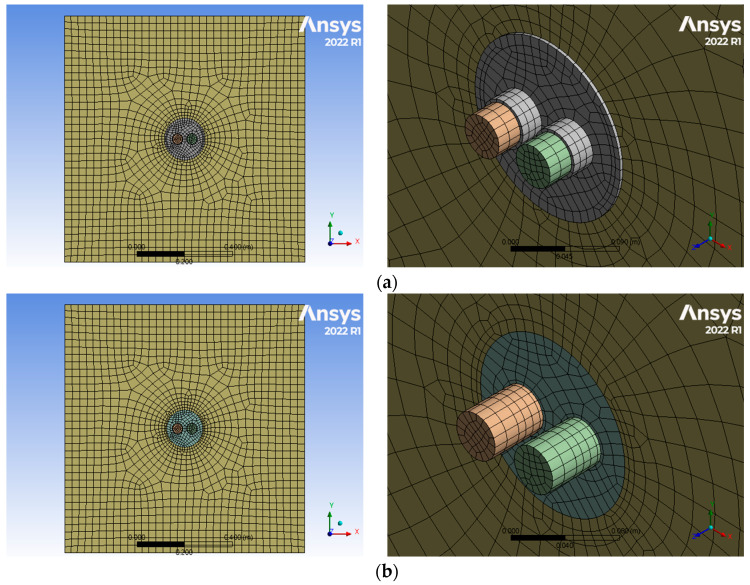
Results of 3D modeling of mixed paint materials. (**a**) 3D model for with mixed paint. (**b**) 3D model for without mixed paint.

When the mass fraction of expandable graphite is 0.5 wt.%, the 3D model created using the input data of the mixed paint is shown in Figure 10.

**Figure 11:**
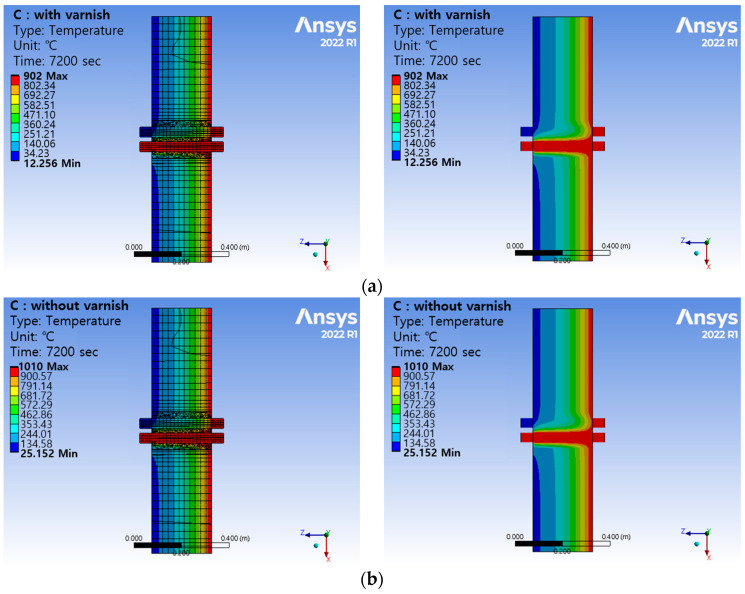
Results of temperature change on the surface after 2 h (simulation). (**a**) Temperature distribution after 2 h (with the paint). (**b**) Temperature distribution after 2 h (without the paint).

**Figure 12:**
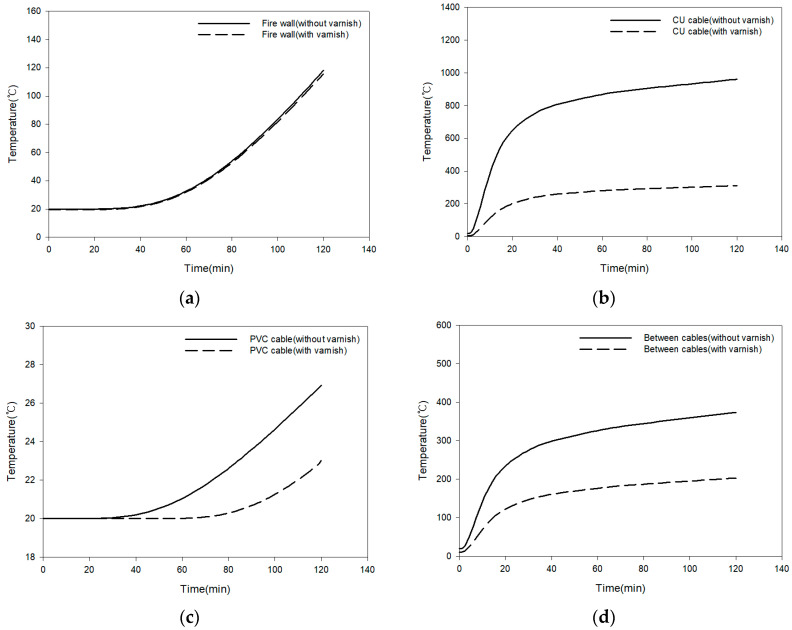
Results of temperature change on the surface after 2 h (graph)**.** (**a**) Fire wall (point 1). (**b**) Cu cable (point 2). (**c**) PVC cable (point 3). (**d**) Between cables (point 4).

The results of the fire simulation performed based on boundary conditions and loading conditions are shown in Figure 11 and Figure 12. Fire simulations were performed under thermal loading conditions. Two hours after the fire broke out, the thermal temperature distribution was analyzed. The temperature distribution was compared with mixed paint and without mixed paint. Two hours after a fire broke out in a virtual space, the approximation value was within ±5% when mixed paint was applied to the firewall (point 1) and when it was not. Since the mixed paint material was not applied to the firewall, it is believed that it did not make a significant difference. In the case of Cu cable (point 2), this is a key area where mixed paint is applied. When the mixed paint was applied, it showed 311.4 °C, and when the mixed paint was not applied, it showed 961.1 °C. When mixed paint was applied to Cu cable, the temperature value decreased by up to 68%. In the case of PVC cable (point 3), the temperature was 23.0 °C when the mixed paint was applied. When there was no mixed paint, the PVC cable part showed 26.9 °C. When the mixed paint was applied between cables (point 4), the temperature value decreased by up to 45%.

A fire model for the firewall penetration area was created. Fire simulation was performed on the created 3D model and the results were analyzed depending on whether mixed paint was applied or not. It was found that when the mixed paint was applied, the surrounding temperature of the penetration area was lower than when the mixed paint was not applied. If research is conducted in parallel with fire simulation technology and actual fire testing, it is believed that it will be possible to reduce the time required for actual fire testing and the cost of fire testing.

## 4. Conclusions

In this research, mixed paints were manufactured by setting different mass fractions of expandable graphite. Thermal, physical, and morphological properties were analyzed according to the mass fraction of expandable graphite. After creating a 3D model for the “firewall penetration part” using the results of thermal property analysis, a fire simulation study was conducted with and without mixed paint materials. The research results are as follows.

The mixed paint with a mass fraction of expandable graphite of 0.5 wt.% had the best thermal properties. When the mass fraction of expandable graphite was 0.7 wt.%, thermal properties decreased. Because the mass fraction of expandable graphite is relatively high, it is believed that agglomeration between particles occurred and the total amount of heat release increased.

As a result of analyzing the physical properties according to the mass fraction of expandable graphite, tensile strength and elongation tended to increase as expandable graphite was added when the amount was 0.5 wt.% or less. When the mass fraction of expandable graphite was 0.7 wt.%, the value decreased by 15.6% compared to when the mass fraction of expandable graphite was 0.5 wt.%. Similar to the results of the thermal property analysis, it is believed that agglomeration occurred between the expandable graphite particles, thereby reducing the physical properties.

As the mass fraction of expandable graphite increased, the number of inorganic particles observed per unit area tended to increase. When the mass fraction of expandable graphite was 0.7 wt.%, the particles were not distributed uniformly due to agglomeration between particles.

Two hours after the fire broke out, the thermal temperature distribution was analyzed. The temperature distribution was compared with and without mixed paint. Two hours after a fire broke out in a virtual space, when mixed paint was applied to the Cu cable, the temperature value decreased by up to 68%. When mixed paint was applied between the Cu cable and PVC cable, the temperature value decreased by up to 45% or more. It was found that when the mixed paint was applied, the surrounding temperature of the penetration area was lower than when the mixed paint was not applied. Through fire simulation technology, the cost and time required for research and development can be dramatically reduced. In the event of an actual fire, paint with high fire resistance can reduce casualties and property damage. We also plan to conduct experiments on the fire resistance of paint in a large-scale fire environment.

## Figures and Tables

**Table 1:** Mixing conditions for high-elasticity paint materials.

Sample	Ingredient	Ratio (%)	Sample	Ingredient	Ratio (%)
#1	Acrylic emulsion binder	90.0	#4	Acrylic emulsion binder	89.5
	Polyurethane dispersion	9.9		Polyurethane dispersion	9.9
	Emulsifier	0.1		Emulsifier	0.1
	Expandable graphite	0		Expandable graphite	0.5
	Sum	100		Sum	100
#2	Acrylic emulsion binder	89.9	#5	Acrylic emulsion binder	89.3
	Polyurethane dispersion	9.9		Polyurethane dispersion	9.9
	Emulsifier	0.1		Emulsifier	0.1
	Expandable graphite	0.1		Expandable graphite	0.7
	Sum	100		Sum	100
#3	Acrylic emulsion binder	89.7			
	Polyurethane dispersion	9.9			
	Emulsifier	0.1			
	Expandable graphite	0.3			
	Sum	100			

**Table 2:** Basic properties of expandable graphite.

Material	Mesh (μm)	Expansion Rate (at 950 °C)
NA.23 (expandable graphite)	60~65	550

**Table 3:** Results of flammability analysis according to mass fraction of expandable graphite.

Test Sample	Ignition (s)	Flame Out (s)	THR (MJ/m^2^)		Mass Loss Rate (%)
			5 min	10 min	
#1	33.2 (±0.8)	70.0 (±0.5)	4.27 (±0.1)	6.42 (±1.1)	8.37 (±1.2)
#2	47.0 (±1.2)	90.5 (±0.8)	1.81 (±0.2)	2.55 (±0.3)	6.31 (±0.2)
#3	48.2 (±0.5)	91.2 (±0.9)	1.52 (±0.1)	2.18 (±0.5)	5.82 (±0.3)
#4	50.5 (±0.3)	95.0 (±1.0)	1.21 (±0.2)	1.82 (±0.2)	4.54 (±0.2)
#5	50.3 (±0.2)	93.5 (±1.2)	1.35 (±0.1)	1.87 (±0.3)	4.87 (±0.1)

**Table 4:** Input data for creating a 3D fire model.

Properties	Input Data				
	Fire Wall	Polyurethane Pad	Mixed Paint	Copper Cable	PVC Cable
Thermal conductivity coefficient (W/m·K)	2.9	7.5 × 10^−2^	5.6 × 10^−2^	396.7	1.8 × 10^−1^
Specific heat capacity (at 298.15 K) (J/kg·K)	936.3	60.1	44.8	383.3	1049

## Data Availability

The data presented in this study are available from the corresponding author upon reasonable request.
